# Molecular Evidence of Demographic Expansion of the Chagas Disease Vector *Triatoma dimidiata* (Hemiptera, Reduviidae, Triatominae) in Colombia

**DOI:** 10.1371/journal.pntd.0002734

**Published:** 2014-03-13

**Authors:** Andrés Gómez-Palacio, Omar Triana

**Affiliations:** Grupo BCEI, Universidad de Antioquia UdeA, Medellín, Colombia; Universidad Autónoma de Yucatán, Mexico

## Abstract

**Background:**

*Triatoma dimidiata* is one of the most significant vectors of Chagas disease in Central America and Colombia, and, as in most species, its pattern of genetic variation within and among populations is strongly affected by its phylogeographic history. A putative origin from Central America has been proposed for Colombian populations, and high genetic differentiation among three biographically different population groups has recently been evidenced. Analyses based on putatively neutral markers provide data from which past events, such as population expansions and colonization, can be inferred. We analyzed the genealogies of the nicotinamide adenine dinucleotide dehydrogenase 4 (ND4) and the cytochrome oxidase subunit 1-mitochondrial genes, as well as partial nuclear ITS-2 DNA sequences obtained across most of the eco-geographical range in Colombia, to assess the population structure and demographic factors that may explain the geographical distribution of *T. dimidiata* in this country.

**Results:**

The population structure results support a significant association between genetic divergence and the eco-geographical location of population groups, suggesting that clear signals of demographic expansion can explain the geographical distribution of haplotypes of population groups. Additionally, empirical date estimation of the event suggests that the population's expansion can be placed after the emergence of the Panama Isthmus, and that it was possibly followed by a population fragmentation process, perhaps resulting from local adaptation accomplished by orographic factors such as geographical isolation.

**Conclusion:**

Inferences about the historical population processes in Colombian *T. dimidiata* populations are generally in accordance with population expansions that may have been accomplished by two important biotic and orographic events such as the Great American Interchange and the uplift of the eastern range of the Andes mountains in central Colombia.

## Author Summary

The Chagas disease vector *Triatoma dimidiata* is one of the most important vectors in America, owing to its wide genetic and epidemiological heterogeneity. Colombian *T. dimidiata* populations occupy eclectic sylvatic ecotopes, but have also been found in dwellings infected with *Trypanosoma cruzi*, and therefore it is considered (along with *Rhodnius prolixus*) the most important vector in several departments. The current study explores the population structure history of Colombian populations by means of a molecular coalescence approach. The results indicate that the historical population processes in *T. dimidiata* in Colombia are in accordance with population expansions that may have been accomplished by two important biotic and orographic events such as the Great American Interchange and the uplift of the eastern range of the Andes Mountains in central Colombia. The genetic history as well as the heterogeneity of the populations could be reflected in different responses of the populations to vector control interventions; thus, a local level of entomological vigilance should be implemented to evaluate the intervention results in each region.

## Introduction

A population's demographic history as well as phylogeographic inferences are usually accessed by studying the reconstructed genealogical histories of individual genes (gene trees) sampled from different populations [1]–[3]: Studying patterns of genetic variation in a geographical context via gene trees can contribute considerably to our understanding of what factors have influenced geographical population structure and species divergence [4], [5]. Coalescent theory [6] is applied to studies relating to the haplotype frequency, genealogy, and geographical distribution of populations, and has been applied as a useful focus for understanding many events that may have occurred in the past across the demographic history of populations (e.g., population expansion, bottlenecks, vicariance, and migration).

*Triatoma dimidiata* is considered the major vector of Chagas disease in several Central American countries as well as in various regions of Ecuador and Colombia [7]. Across its distribution in Colombia, *T. dimidiata* occupies a great diversity of habitats, including sylvatic habitats such as palm trees and hollow trees in northern regions, or rock piles, as well as intradomiciliary synanthropic habitats mostly in the country's central Andean departments [8]. Previous studies have suggested that *T. dimidiata* shows a strong and significant genetic structure related to its original eco-geographical regions in Colombia [9], which, albeit weakly, correlates with an isolation-by-distance model [10]. A preliminary paper on the genetic diversity and population differentiation of *T. dimidiata* in Colombia was assessed using DNA sequence analysis of the nicotinamide adenine dinucleotide dehydrogenase 4 (ND4) mitochondrial gene, which interestingly suggested a high genetic interpopulation differentiation within Colombia [9]. However, because the sample evaluated was rather small (*n* = 40), representing only a minimal area of the species distribution, a more exhaustive genetic analysis of several communities of Colombian *T. dimidiata* was performed by using a microsatellite as well as cytochrome c oxidase subunit 1 (CO1) gene [10]. Here, three major clusters with distinct ecological attributes were distinguished. These three clusters were termed: (i) Inter-Andean Valleys (IAV), harboring a population group located in central Colombia, where *T. dimidiata* shows more epidemiological relevance and apparently high flow between synanthropic and sylvatic habitats; (ii) the Caribbean Plains (CP) population group, the most widely distributed group from the Caribbean coast to the lowlands of the Central Andean Cordillera, occupying mainly sylvatic habitats; and (iii) the Sierra Nevada de Santa Marta (SNSM) mountain population group, located in the northwestern zone of Colombia, occupying exclusively sylvatic habitats such as palm trees, although a few individuals have also been found sporadically visiting indigenous dwellings and have also been implicated in human *Trypanosoma cruzi* infections [11], [12].

In a phylogeographic context, according to the evidence addressed by molecular analyses of ITS-2 [13], cytochrome b (cyt b), and ND4 genes [14], Colombian *T. dimidiata* populations are considered a differentiated form derived from Central American conspecific populations (in fact, it might be considered an additional subspecies or species within *T. dimidiata sensu lato*, according to the authors) [7], [13]–[16]. Under this hypothesis, Colombian populations are thought to have originated from an ancient population introduced through the Isthmus of Panama [13] after its emergence between 1.9 and 3.8 mya [14], therefore undergoing a wide geographical expansion at a later time that gave birth to the current population structure. Consequently, the aim of the present study was to assess the population structure and history as factors explaining the geographical distribution of population groups of *T. dimidiata* in Colombia as well as their position in the phylogeographic picture proposed for the species so far.

Analysis of the population genetics in the context of the geographic structure suggests demographic processes that occurred in the past. Thus, while the pattern of variation in mtDNA haplotypes allows one to identify geographical distribution differentiation among groups of haplotypes in several populations, it also supports inferences on demographic events that occurred in the past, such as geographical range expansion and population size, according to coalescence theory. In this way, the change in population size through genealogy is reflected by a haplotype network with a star shape [17], an excess of rare mutations resulting in an excess of low-frequency haplotype presence [18], and a unimodal mismatch nucleotide distribution [19], [20].

In this study, we broaden the knowledge of the spatial structure of the three population groups of *T. dimidiata* in Colombia by analyzing ND4 gene nucleotide sequences obtained from 228 specimens in 22 localities; subsequently, several historical demographic tests were conducted using ND4 combined with previously reported CO1 nucleotide sequences [10] and ITS-2 rDNA. Finally, we also explored the phylogeographic pattern of Colombian populations by including the available ITS-2 and ND4 sequences of Central American and Mexican conspecific populations.

Knowledge of population dynamics issues such as geographical dispersion and individual migration between extradomiciliary and domiciliary ecotopes is essential for predicting the success of vector control and surveillance strategies against Chagas disease. In this sense, study of the population structure and demographic history in the most relevant vectors is required for the design of more effective intervention strategies.

## Materials and Methods

### Insect samples

**Figure 1:**
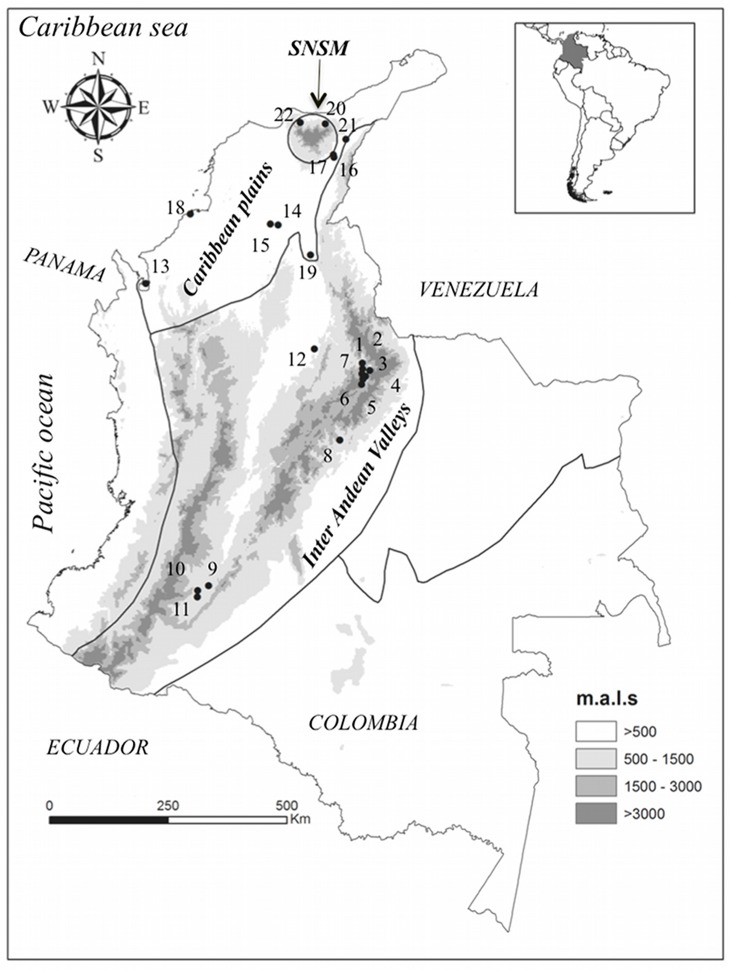
Geographical distribution of the sampling sites of the Colombian *T. dimidiata* populations studied. Map of Colombia showing the 22 *T. dimidiata* sampling sites used in differentiation and demographic analyses. Site numbers are detailed in Table 1. Eco-geographical regions correspond to the Inter-Andean Valleys region, the Caribbean Plains region, and the Sierra Nevada de Santa Marta Mountain.

A total of 228 sequences for the ND4 (624-bp) gene as well as 42 partial sequences (252 bp) for ITS-2 were obtained (Table 1). Individuals were collected in intradomiciliary, peridomiciliary, and sylvatic ecotopes of 22 municipalities from ten departments in Colombia (Table 1 and Figure 1). Bug captures were carried out in 2003–2009 in collaboration with local personnel from the Ministry of Health. Sylvatic samples were collected with live-baited traps [21]. Domiciliary and peridomiciliary collections were made using the traditional manual collection method using a dislodging spray [22] and capture by homeowners. Captures from palm trees were obtained through palm dissection as described elsewhere [23], with consent previously obtained from the landowners. All specimens were identified to the species level using Lent and Wygodzinsky's typological key [24] and kept in 70% ethanol until being processed for DNA extraction.

**Table 1:** Origins and number of Colombian *T. dimidiata* samples used.

Region	Map No.	Department	Sampling sites (locality)	Longitude	Latitude	Altitude (masl)	Ecotope	ITS-2	ND4	CO1‡
**Inter-Andean Valleys**	1	Boyacá	Soata*, Urban zone, El Espino	−73.1300	5.0800	2501	I	0	13	2
	2		Soata, La Costa	−72.7100	6.3333	1710	P, S	0	19	9
	3		San Mateo*, Huerta Vieja, Guayabal	−72.5643	6.3927	2177	I	1	6	4
	4		Boavita*, Espigón, Lagunillas	−72.6412	6.2838	1479	I	1	2	2
	5		Susacon, Urban zone	−72.6900	6.2300	2488	I	0	1	1
	6		Tipacoque, Urban zone	−72.6953	6.4233	2020	I	2	12	3
	7		Sativanorte*, Baracuta, La estancia, Datal	−72.7114	6.1344	2813	I	3	6	1
	8		El Espino, Urban zone	−72.4959	6.4833	2148	I	2	4	0
	9	Huila	Pital, San Joaquin	−75.8186	2.2306	976	I	0	1	0
	10		Tarqui, Urban zone	−75.8203	2.1114	828	I	0	2	1
	11		Gigante, Veracruz	−75.6101	2.3233	754	I	0	1	1
	12	Santander	Capitanejo, Chorreras	−72.7003	6.5314	1086	I, P	3	23	6
**Caribbean Plains**	13	Antioquia	Turbo, Blanquiset, Villa Eugenia	−76.9338	7.8745	6	S	3	5	2
	14	Bolívar	Margarita, Urban zone	−74.2928	9.1457	20	S	3	2	4
	15		San Fernando, Urban zone	−74.4385	9.1653	18	S	0	9	2
	16	Cesar	Valledupar*, Armawake, Tamacal	−73.2392	10.4275	132	P	2	11	5
	17		Valledupar*, Seyminin, Bechungaka, Sabana de Crespo, Donachui, Chemesquemena	−73.2506	10.4769	176	I	5	47	11
	18	Córdoba	San Bernardo del Viento, Playas del viento	−75.9558	9.3558	9	S	2	1	0
	19	Norte de Santander	El Carmen*, Tierra Azul, Zaragosa, El Chamizón, Santa Rita, Maracaibo	−72.8708	8.0308	714	I	7	19	3
**Sierra Nevada de Santa Marta**	20	La Guajira	Dibulla*, Gumake, Umandita, Taminaca	−73.2940	11.1781	365	S	2	13	8
	21		San Juan del Cesar*, Marocazo, Ulago, Cherua	−73.0133	10.8112	423	I	4	11	9
	22	Magdalena	Santa Marta*, Guachaca-Cacahualito, Tarapaca, Mendihuaca	−73.8219	11.2117	283	S	2	20	12
**Total**								**42**	**228**	**86**

Number and geographical origin of Colombian *T. dimidiata* samples included in the study. Ecotopes: I, intradomiciliary; S, sylvatic; P, peridomiciliary.

* Insects from nearby localities were grouped as one sampling site.

‡ Sequences reported in [10].

### DNA extraction, PCR amplification, and sequencing

Genomic DNA was obtained from four legs of each insect or from the thorax muscle when necessary (i.e., in old, dead, dry bugs, or those that had lost their legs). DNA extraction was performed according to a previously reported mosquito DNA-extraction protocol [25]. For each specimen, a 614-bp fragment of the ND4 gene was PCR-amplified using ND4-F (5′-TCAACATGAGCCCTTGGAAG-3′) and ND4-R (5′-TAATTCGTTGTCATGGTAATG-3′) primers [9]. PCR reactions for the mitochondrial gene were conducted in a final volume of 35 µl using a 30-ng DNA template, 1× PCR buffer (0.1 M Tris-HCl, 0.5 M KCl, and 0.015 M MgCl_2_, pH 8.3), 250 µM dNTP, 0.016 µM of each primer, 5 mM MgCl_2_, and 2 U of Taq DNA polymerase (Promega®). The fragments were amplified with the following thermal cycling conditions: 95°C for 5 min; 35 cycles of 94°C for 30 s, 50°C for 30 s, and 72°C for 60 s; 72°C for 10 min.

Because no reproducible and unspecific amplifications in Colombian *T. dimidiata* specimens were obtained when universal primers 5.8S and 28T [16] were used, we employed the *T. dimidiata*-specific primers TdITSF (5′-TGGAAATTTTCTGTTGTCCACA-3′) and TdITS-2R (5′-CTTGCTTTATACAACAAGAAGTA-3′) [26] for PCR amplification of a 252-bp fragment of ITS-2 rDNA. PCR reactions were conducted in a final volume of 35 µl using 30-ng DNA templates, 1× PCR buffer (0.1 M Tris-HCl, 0.5 M KCl, and 0.015 M MgCl_2_, pH 8.3), 250 µM dNTP, 0.025 µM of each primer, 3 mM MgCl_2_, and 2 U of Taq DNA polymerase (Promega®). After an initial denaturation of 95°C for 5 min, PCR reactions comprised 35 cycles at 95°C for 30 s, 60°C for 30 s, and 72°C for 30 s, followed by a final extension of 72°C for 7 min [26]. All PCR products were sent to Macrogen Inc., Seoul, Korea, for DNA purification and sequencing service. For all samples, sequencing was conducted in both forward and reverse directions.

### Sequence analyses and inter-population differentiation

Forward and reverse sequences from specimens were used to generate a consensus sequence with a previous pairwise alignment using the CLUSTALW algorithm [27] implemented in Bioedit v. 7.0.5 [28]. Posterior multiple sequence alignment for each DNA marker was performed using the CLUSTALW algorithm [27].

In the complete data set for ND4 and ITS-2, we evaluated the nucleotide diversity (π), number of haplotypes (h), and haplotype diversity (Hd) using DnaSP v.5.10 [29]. The genetic differentiation among Colombian geographical samples was assessed by Fst comparison, and both nucleotide and haplotype diversity levels were estimated using Hudson's statistics Kst and Hst [30], defining the statistical significance (*p*<0.001) with a permutation test of 1,000 replicates. A median joining (MJ) haplotype network was used to examine inter-haplotype relationships among the 155 haplotypes of the 228 ND4 sequences as well as for 17 partial ITS-2 haplotypes using default parameters in Network 4.6.0.0 software (http://www.fluxus-engineering.com).

### Spatial inter-population structure

Spatial analysis of molecular variance (SAMOVA) was performed to estimate the structure among population groups according to pairwise geographical distances between geographical locations by Fct statistical calculations using SAMOVA v.1.0 [31]. Fct values were estimated for simulated population groups from k = 2 to k = 4 in 1,000 iterations of the data set, which corresponds to the number of eco-geographical regions of *T. dimidiata* populations suggested in Colombia plus or minus one. The maximized Fct was selected according to the highest significant (*p*<0.001) value.

An interpolation-based graphical method was employed to generate a three-dimensional genetic landscape shape (GLS) within the Alleles in Space (AIS) program [32]. This analysis provides a visual perspective of the spatial distribution of the genetic structure over landscapes, with peaks in areas where pairwise genetic distances between haplotypes from each geographical location are high, and valleys where genetic distances between individuals are low (the x- and y-axes represent latitude and longitude, whereas the z-axis represents genetic distances) [32]. Georeferenced coordinates (Universal Transverse Mercator system) were provided for each individual and analyzed for the ND4 sequences. Additionally, we performed a spatial autocorrelation analysis to test whether there were significant correlations (based on Vendramin et al. [33] correlation index V) between average pairwise genetic distances of haplotypes (Ay) in each spatial class defined according to geographical distances among geographical locations (y). This analysis is illustrated by a distogram where Ay takes on a value of 0 when all individuals within distance class y are genetically identical, and takes on a value of 1 when all individuals in distance class y are completely dissimilar. Spatial autocorrelation analysis was performed on 10 spatial classes with unequal distance and equal sample size (approximately 20 observations per class) in the AIS software [32]. Likewise, to test whether the inter-geographical location structure fits an isolation by distance model, we performed a Mantel test [34] on the pairwise genetic and geographical distance matrices, and the statistical significance (*p*<0.001) was assessed by a permutation test of 1,000 replicates.

### Molecular analysis of population history and timing

The demographic history of the Colombian *T. dimidiata* was investigated by comparison of mismatch distributions of pairwise nucleotide differences under an expected constant and fluctuating population size in 10,000 generations of coalescent simulations using DNaSP v. 5 [29]. The distribution of mismatch pairwise nucleotide differences was obtained for ND4 haplotypes, as well as combined with CO1 haplotypes (*n* = 86; sequence size, 1,016 bp), and for partial ITS-2 sequences assuming free recombination. Parameters for a sudden demographic expansion were estimated using the sum of squares deviation (SSD) [35] and Harpending's raggedness index (Rag) [36] implemented in Arlequin v 3.1 [37]. Tests for neutrality were also assessed for access to the demographic history. Fu's F_s_ [38] and the Ramos-Onsins and Rozas R_2_ [39] statistics for detecting population growth were estimated under coalescent simulations with 10,000 generations using DNAsp v.5 software [29].

To visualize the effective breeding population size (Ne) fluctuation over time, a Bayesian skyline plot (BSP) analysis [40] was performed as implemented in the BEAST 1.6 package and Tracer v1.5.1 [41]. The starting trees were tested initially for Colombian *T. dimidiata* using ND4 haplotypes, and combined ND4 and CO1 genes, and then tested for additional haplotype sequences of Central American and Mexican conspecifics for the ND4 (*n* = 39) gene and ITS-2 (*n* = 66; Table S1). Trees were obtained using the maximum likelihood (under GTR+G) substitution model after checking according to the Akaike criterion [42] implemented in the jModelTest software [43] with an uncorrelated lognormal relaxed molecular clock assuming one generation per year, as reported for Colombian *T. dimidiata* individuals [44]. Two separate runs of BMCMC were performed, and a simulated population size (ESS) greater than 200 was obtained using a chain length of 10×10^6^, assuming 10 stepwise control points as the number of coalescent groups.

### Phylogenetic relationship

The complete data set of ITS-2 (*n* = 83) and ND4 (*n* = 194) haplotypes including Central American and Mexican conspecifics was used to perform a Bayesian inference (BI) Markov Chain Monte Carlo (BMCMC) approach as implemented in the BEAST v.1.6.1 package [41]. The topologies were inferred from the GTR+G substitution model and the model parameters (base frequencies, transition/transversion ratio, rate variation shape parameter) were derived empirically. Metropolis coupling was used with two chains per analysis. BMCMC was run for 10×10^6^ generations, with a sampling frequency of 1,000. Two independent trees were combined using LogCombiner v.1.6.1, and convergence of parameters in the Bayesian analyses was assessed with Tracer v.1.5, after discarding a 10% burn-in. Finally, a majority rule consensus tree (>0.75 posterior probability node support) was calculated from all trees sampled using TreeAnnotator v.1.6.1 in the BEAST v.1.6.1 package [41].

Additionally, a maximum likelihood (ML) tree was estimated using the GTRCAT approximation of substitution model, and the best knowledge likelihood tree (BKLT) was selected via bootstrapping (10,000 replicates) in RAxML-VI-HPC v.2.2.3 [45]. ML tree nodes showing bootstrap support of more than 75% were considered as well supported. Topologies were edited with the FigTree v.1.3.1 software (http://tree.bio.ed.ac.uk). The overall topological match score and a well-supported node match score between IB and ML topologies for both ND4 and ITS-2 markers were calculated using Compare2Trees software [46].

### Accession Numbers

All nucleotide sequences are available with GenBank accession codes for ND4: KC489309–KC489463 and ITS-2: KC489292–KC489308.

## Results

### Genetic differentiation and geographical structure

The ND4 gene analysis at both haplotype and nucleotide diversity levels showed a statistically significant differentiation index among the three eco-geographical groups (Kst = 0.235; *p*<0.001 and Hst = 0.0166; *p*<0.001). Moreover, the ITS-2 marker indicated low variability, mostly in the SNSM region (Table 2). From this result, we consider that ND4 offers a better resolution for exploring the spatial structure of Colombian *T. dimidiata* populations, and therefore ITS-2 was excluded from these analyses. Thus the overall Fst value (0.482; *p*<0.05) using ND4 indicates high genetic differentiation among eco-geographical groups. Pairwise Fst was 0.592 (*p*<0.05) between CP and SNSM, 0.588 (*p*<0.05) between IAV and CP, and 0.512 (*p*<0.05) between IAV and SNSM.

**Table 2:** Gene diversity and differentiation between eco-geographical regions of Colombian *T. dimidiata*.

Gene diversity										
ND4						ITS-2				
Region	N	n	π (± SD)	h	Hd (± SD)	N	n	π (± SD)	h	Hd (± SD)
Inter-Andean Valleys	12	90	0.025 (0.002)	79	0.996 (0.003)	6	12	0.011 (0.002)	9	0.939 (0.003)
Caribbean Plains	7	94	0.016 (0.002)	48	0.959 (0.011)	6	22	0.006 (0.002)	8	0.649 (0.111)
SNSM	3	44	0.023 (0.006)	30	0.962 (0.019)	3	8	0.001 (0.000)	2	0.250 (0.180)
**Total**	**22**	**228**	**0.041 (0.001)**	**155**	**0.991 (0.002)**	**15**	**42**	**0.007 (0.001)**	**17**	**0.702 (0.080)**

Haplotype-based (Hst)/Nucleotide-based (Kst) differentiation statistics									
	Inter-Andean Valleys	Caribbean Plains		SNSM	Inter-Andean Valleys		Caribbean Plains		SNSM
Inter-Andean Valleys		0.011*		0.009*			0.042 ns		0.101 ns
Caribbean Plains	0.418*			0.017*	0.101 ns				0.001 ns
SNSM	0.318*	0.388*			0.103 ns		0.014 ns		

Summary of genetic diversity indices and pairwise comparison based on haplotype (Hst above diagonal) and nucleotide diversities (Kst below the diagonal) of the ND4 (614-bp) gene and partial sequences of ITS-2 (252-bp) in *T. dimidiata* from several regions in Colombia. Notations: N: number of localities; n: number of ND4 sequences; π (±SD): nucleotide diversity (SD); h: number of haplotypes and Hd (±SD): haplotype diversity (SD); ns: not significant and *p<0.001.

**Figure 2:**
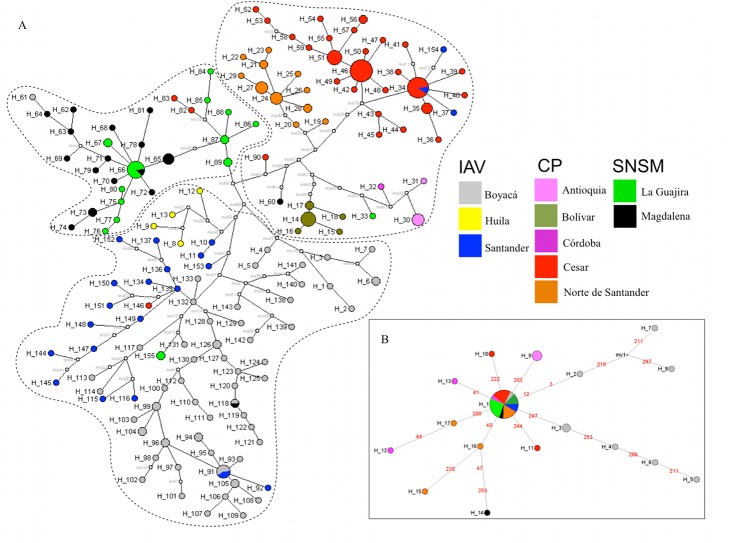
Median-joining haplotype network of the ND4 gene and partial ITS-2 rDNA of *T. dimidiata* in Colombia. A) ND4 and B) ITS-2 haplotypes found in the study (Tables S3 and S2, respectively) were included in a network analysis according to the eco-geographical region of origin (Table 1). Haplotype frequency is represented by the size of each node and white nodes represent hypothetical haplotypes (or median vectors). Sampled sites were assigned different colors as coded in the legend.

A large number of unique ND4 haplotypes (h = 155), which are likely to be rare or recent in a population, were widely distributed in the haplotype network according to the three eco-geographical groups suggested (Figure 2A; [10]), whereas for partial ITS-2 haplotypes (h = 17; Table S2) a clear star-shaped network with no differentiation among haplotypes from a particular eco-geographical region was observed (Figure 2B). Despite large numbers of hypothetical haplotypes (median vectors that suggest both unsampled or extinct haplotypes) observed in the ND4 network, those haplotypes from sampled sites of each eco-geographical region were closely related in almost all cases (Figure 2A and Table 1). Out of 155 ND4 haplotypes found (Table S3), only 10 were apparently more closely grouped with an unassigned eco-geographical region than indicated, suggesting possible gene flow among regions or retained haplotypes of an ancient origin (Table 1 and Figure 1).

**Figure 3:**
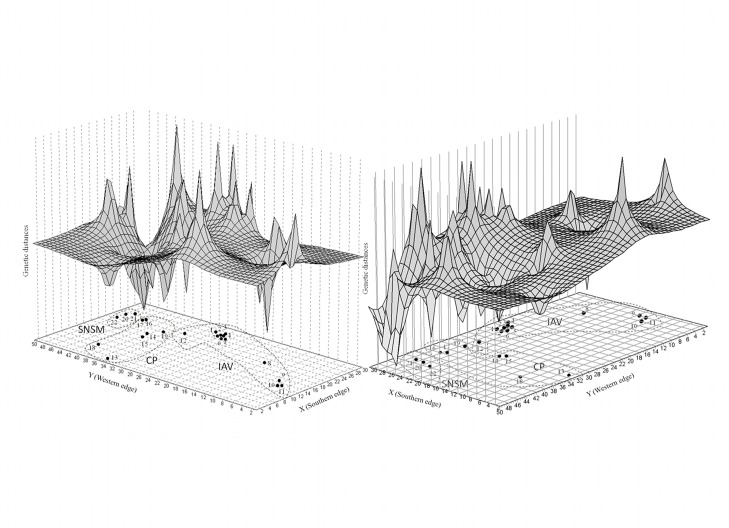
Landscape shape interpolation of genetic distances within *T. dimidiata* in geographical space in Colombia. A graphical interpolation-based representation of the ND4 gene's genetic diversity was made using a 30×50 grid and a distance-weighting parameter of 1 for *T. dimidiata* in Colombia. The x- and y-axes represent geographical coordinates, while surface heights along the z-axis indicate genetic distance. Peaks and valleys are indicative of areas with high or low pairwise genetic distance between ND4 haplotypes, respectively. The position of sampling sites in the “base” of the graph is the approximate distribution of localities described in Table 1 and Figure 1.

Spatial analysis of molecular variance performed to assess the substructure within *T. dimidiata* indicated a significant maximized Fct at k = 3 (0.485; *p*<0.001), supporting the eco-geographical structure previously evidenced. Collection sites from the Boyacá, Santander, and Huila departments comprised the first group (congruent with the IAV region), sites from Bolívar, Antioquia, Norte de Santander, Córdoba, and Cesar formed the second group (congruent with the CP region), and Magdalena and La Guajira sites formed the third (the SNSM region). Genetic landscape shape interpolation analysis showed that the spatial distribution of haplotype diversity across Colombia was not uniform, as indicated by the presence of peaks and valleys (Figure 3). The lowest pairwise genetic distances in *T. dimidiata* geographical locations were detected across the CP region, and the highest in both IAV and SNSM (Figure 3).

**Figure 4:**
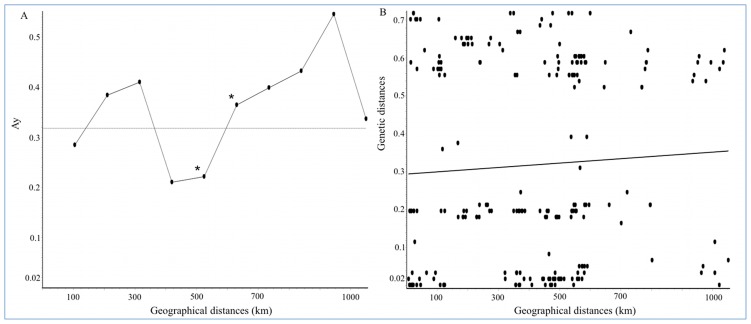
Distance-based analyses of population structure of *T. dimidiata* in Colombia. A) Distogram of spatial autocorrelation analysis of *T. dimidiata* from Colombia showing the relationship between pairwise genetic ND4 gene distances (y-axis) and geographical distances (m) in 10 spatial classes (x-axis). Horizontal line indicates the average value of Ay for the data set. Asterisk represents a significant (*p*<0.05) Miller autocorrelation index (Ay). B) Mantel test of genetic (ND4 gene) and geographical distances (m) between the geographical locations of *T. dimidiata* analyzed in this study.

Spatial autocorrelation analysis showed a low and nonsignificant autocorrelation value for the full data set (V = 0.01; *p*>0.05), suggesting that nonsignificant clustering of the haplotypes (based on pairwise genetic distance) within each of the 10 spatial classes tested could be inferred (for instance, genetic distances between haplotypes from a spatial class 100 m apart is not significantly lower than estimated between haplotypes ∼425 m apart, as expected under an isolation-by-distance model, see Figure 4A).

The bimodal shape observed in the distogram intersect the mean genetic distance value (0.032) around the central geographical distances of the classes, and showed a significant autocorrelation when spatial classes ranking between ∼525 m and ∼625 m (Figure 4A). This results indicates that pairwise genetic differences can be higher than average at both shorter and longer geographical distances, and lower at moderate geographical distances (Figure 4A). Furthermore, genetic distances between haplotypes separated about ∼525 m are significantly lower than those haplotypes separated by ∼625 m (Figure 4A). These results could reflect the geographical range of gene flow among the three eco-geographical population groups described, although further specific analyses of intra- and inter-regional gene flow must be performed. Spatial autocorrelation analysis results were congruent with those of Mantel's test, in which the correlation index between genetic and geographical distances among individuals of the geographical locations analyzed was not significant (r = 0.098; *p*>0.05), indicating no isolation-by-distance model fit that could explain the geographical structure of *T. dimidiata* in Colombia (Figure 4B).

### Inferences drawn from the population history

**Figure 5:**
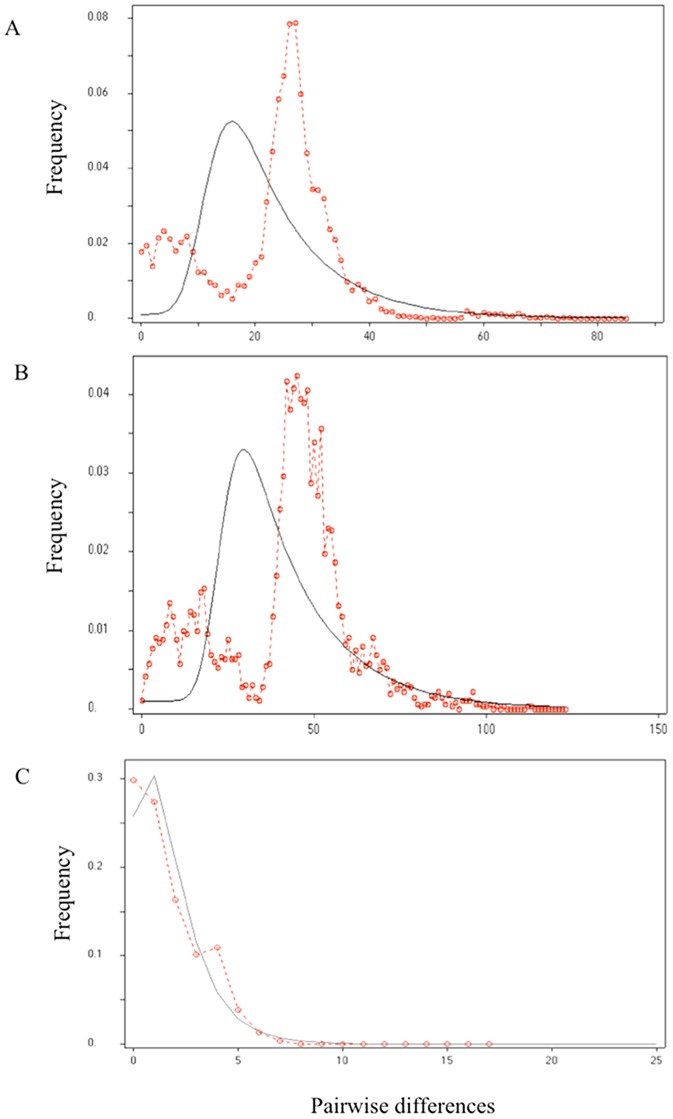
Mismatch distribution among *T. dimidiata* haplotypes of Colombia based on A) ND4; B) ND4+CO1 genes, and C) partial ITS-2 rDNA. Expectations from the stepwise growth model are superimposed (black lines) on the observed values (red line).

The mismatch distribution of ND4, ND4+CO1, and of partial ITS-2 rDNA were fit to expect a mismatch distribution with a fluctuating population size (Figure 5). The goodness of fit of the mismatch distribution between the observed and expected results – under population expansion – was identified (**ND4**: *Rag* = 0.003, *p*>0.05; SSD = 0.003, *p*>0.05; **ND4+CO1**: *Rag* = 0.001, *p*>0.05; SSD = 0.003, *p*>0.05; and **ITS-2**: *Rag* = 0.019, *p*>0.05; SSD = 0.002, *p*>0.05). Thus, the possibility that the expansion model fits cannot be rejected. In addition, significant values for F_s_ and R_2_ in the neutrality tests used for detecting population expansion were found in the three sequence data sets, indicating clear signals of population growth in *T. dimidiata* in Colombia (Table 3).

**Table 3:** Diversity and neutrality estimates for ND4, combined ND4 and CO1 genes, and partial ITS-2 of Colombian *T. dimidiata*.

	ND4	ND4+CO1	ITS-2
**h**	155	86	17
**S**	234	285	16
**k**	25.4	41.67	1.63
**Hd (± SD)**	0.991 (0.002)	0.998 (0.002)	0.702 (0.081)
**θ (± SD)**	0.066 (0.015)	0.043 (0.014)	0.015 (0.003)
**π (± SD)**	0.041 (0.001)	0.041 (0.002)	0.066 (0.001)
**F_s_**	−197.6*	−41.76*	−13.29*
**R_2_**	0.07*	0.09*	0.12*

Notations: h: number of haplotypes; S: number of segregating sites; k: average of pairwise nucleotide differences; Hd (±SD): haplotype diversity (SD); θ (± SD): nucleotide polymorphism (per site); π (±SD): nucleotide diversity (SD); F_s_: Fu's F_s_ [38] neutrality test; R_2_: Ramos-Onsins and Rozas [39].

*Significant at the *p*<0.05 level after coalescent simulations (10,000 replicates).

**Figure 6:**
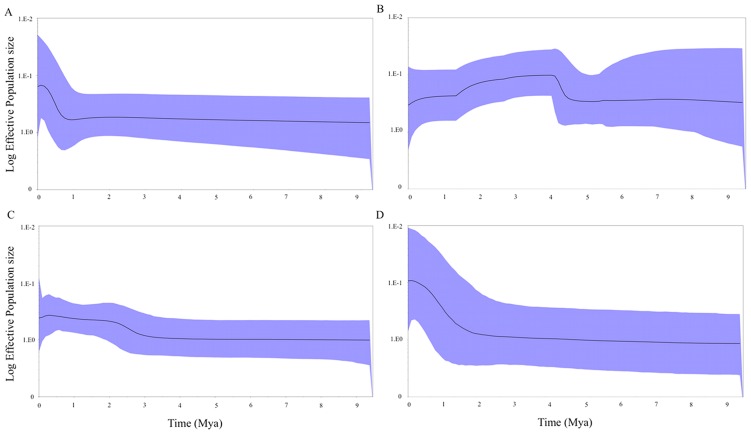
Bayesian skyline plots showing *T. dimidiata* population size changes over time. The relative population size measured as a product-effective population size (y-axis) is shown over time in millions of years (x-axis) in simulated coalescent-based genealogies using A) ND4 Colombian haplotypes, B) combined ND4 and CO1 Colombian haplotypes, C) ND4, and D) partial ITS-2 haplotypes including Central American and Mexican conspecific sequences (Table S1). The thick black line is the median estimate and the solid (blue) interval shows the 95% highest posterior density limits.

The Bayesian skyline plot (BSP) of ND4, and combined ND4 and CO1 genes, indicated that *T. dimidiata* in Colombia seems to have gone through an effective population increase ranging from 1 to ∼4 mya before the present (Figure 6A and 6B). Moreover, the BSP for the ND4 gene including Central American and Mexican isolates supported the suggested population increase approximately 2 to 3 mya before the present (Figures 6C), and the BSP for partial ITS-2 including Central American and Mexican isolates indicated that the population increase was approximately 1.5 mya (Figure 6D).

### Phylogenetic analysis

**Figure 7:**
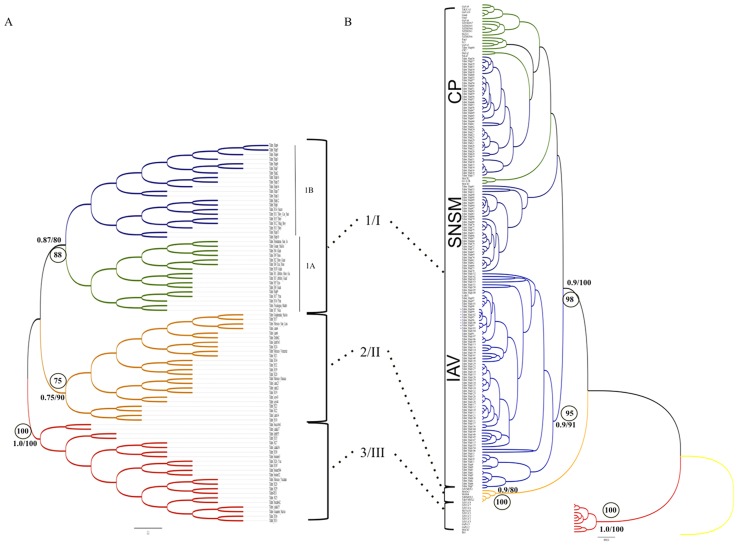
Phylogenetic analyses of *T. dimidiata* based on A) partial ITS-2 fragment and B) the ND4 gene. Numbers on nodes indicate posterior probability and bootstrap values (only posterior probabilities >0.75 and Bootstrap values >75% are shown); circled numbers indicate match node scores between BI and ML-based topologies. In B) the names of the three eco-geographical regions described for Colombian *T. dimidiata* are indicated. Branch color codes and dotted lines indicate each of the main different genetic groups that comprise the *T. dimidiata* species complex according to Bargues *et al.*, (2008) and Monteiro *et al.*, (2013).

The overall topological match score between IB and ML approaches was moderate for both ND4 (65%) and ITS-2 (49.6%), but high match node scores (>75%) were obtained between them for the well-supported clades observed using both markers (Figure 7). This result indicates incomplete congruence of phylogenies was obtained by the IB and ML approaches using both ND4 and ITS-2 markers, but high congruence of the monophyletic clades comprising the *T. dimidiata* genetic groups [13], [14].

The topologies built using the partial ITS-2 showed three main clades (Figure 7A), congruent with the previously reported monophyletic clades using a complete ITS-2 sequence, termed groups 1, 2, and 3 [13]. Within group 1, a low node match score between the IB and ML approaches as well as low node support (posterior probability <0.7 and bootstrap values <75%, data not shown) was observed between Colombian and most Central American haplotypes, which were previously suggested as subgroups 1A and 1B, respectively [13]. This indicates that an inconclusive monophyletic status can be assigned to Colombian and Central American isolates.

Moreover, in the ND4 phylogeny four well-supported monophyletic clades showing a high match node score (>95%) were found (Figure 7B). The four clades included haplotypes belonging to the suggested groups III, II, and I [14] plus a secondary clade within group I harboring haplotypes of the Colombian IAV region (Figure 7B).

## Discussion

In this study, we used molecular data of mitochondrial and nuclear genealogies to access the Colombian *T. dimidiata* population history. The geographical structure patterns obtained using mtDNA sequences were congruent with previous results [10]. These results support the suggested eco-geographical structure of *T. dimidiata* expanding into Colombia, but this structure was not related to the isolation-by-distance model explaining genetic differences in the three main population groups. Instead, a strong signal arguing for a sudden change in population size and range expansion undergone in the recent past after the emergence of the Isthmus of Panama (dating between ∼3.5 mya [47] and ∼10 mya [48]) seems to be a better explanation for assessing the high diversity among *T. dimidiata* population groups in Colombia.

The phylogeographic pattern found here is congruent with that reported for the *T. dimidiata* species complex, where Central American populations are grouped in a monophyletic clade with Colombian specimens (termed group 1/I according to Bargues et al., 2008; Monteiro et al., 2013), thus suggesting a possibly Mesoamerican origin of Colombian populations. However, the phylogenetic result obtained with the ND4 gene (Figure 7B), where a secondary clade was found within group I, suggests populations from the IAV region could be understood as an explicit case of local adaptation accomplished with geographical dispersion across this area of Colombia.

A remarkable issue that needs to be studied further is the domiciliation process shown for the IAV populations, differing from the process undergone by the SNSM and CP populations. This issue requires rigorous ecological approaches to help understand the local forces that shaped the biological diversity of the Colombian *T. dimidiata* natural populations.

### Spatial structure and genetic signals of population expansion in Colombian *T. dimidiata*

The results reported herein on the genetic structure among the three eco-geographical regions IAV, CP, and SNSM are in agreement with previous studies discussing several epidemiological considerations [9], but they also provide additional information on the spatial picture of this genetic diversity and its possible origin.

Pairwise comparison between population groups at both the haplotype (Hst) and nucleotide (Kst) diversity levels indicates that the differential distribution of genetic variability is placed among the IAV, CP, and SNSM regions. Additionally, the results of spatial genetic structure analyses (Fct, genetic landscape shape, spatial autocorrelation, and the Mantel test) make at least two main conclusions possible: (i) there is a heterogenic geographical distribution of genetic diversity and (ii) none of the correlations among genetic divergence and geographical distance explains the geographical group structure of a population.

Spatial interpolation of genetic diversity in Colombia shows a bimodal curve-shaped distribution with SNSM and IAV having higher diversities than the CP region, indicating that several microevolutionary processes have been involved in the genetic diversity divergence among population regions, where possible disruptive segregation of ancestral populations of *T. dimidiata* could be one of many probable causes. An additional observation can be made about this distribution. While *T. dimidiata* populations in SNSM and IAV occupy mostly diverse mountain and premountain ecosystems located around 1,000 masl, CP is for the most part composed of extended lowlands with dry and warm zones at lower altitudes, given that the departments of the CP region extend from the Colombia–Panama border to the foothills of the west mountain range of the northern Andes.

The current eco-geographical structure of Colombian *T. dimidiata* cannot be understood separately from its history. Unlike the previous hypothesis on the possible isolation of population groups due to geographical distance explaining the genetic differences identified [10], the present results further suggest that the spatial genetic structure in Colombian populations could be the consequence of a recent (after ∼4 mya) sudden increase in population size and range occurring after the Great American Interchange (>4.5 mya), contemporary with the rapid uplift of the Eastern Cordillera of the Andes mountain range in Colombia (dating between 2 and 5 mya) [49], where local adaptations of populations shaped *T. dimidiata* diversity.

With the emergence of the Isthmus of Panama, an important paleozoogeographical event occurred as a product of the Great American Interchange between North America via Central America to South America and vice versa for fauna during the Piacenzian (or after early Miocene [48]) age. We suggest that as consequence of this process, Central American populations colonized the Colombia–Panama border regions, inhabiting mostly hollow trees and palm trees, spreading across the Colombian Caribbean Coast Plains, SNSM, and IAV. Afterward, the concordance of (1) the allopatric separation between the two main population groups (SNSM-CP and IAV) and (2) the uplift of the Eastern Cordillera of the Andes mountains in Colombia as well as the SNSM mountain areas (dating between 2 and 11 mya), dates the beginning of the eco-geographical structure detected in *T. dimidiata*.

We also note that an alternative phylogeographical hypothesis indicating a northern South American origin could be postulated for the Colombian IAV region *T. dimidiata* populations based on the high genetic diversity. Although Ecuadorian populations are considered to have been passively introduced from Nicaragua [13], [14], southern and central IAV Colombian sylvatic specimens inhabit diverse ecotopes (hollow trees and rock piles) and their high genetic diversity could indicate originating foci for this Colombian population group (or a sibling species according to Monteiro et al., 2013) in the Central Andean Valleys around Colombia. Although an unquestionably close phylogenetic relationship between *T. dimidiata sensu lato* and the phyllosoma species complex from Mexico [16] supports the Central America origin of this species, we consider further hypotheses should not be completely disregarded. Moreover, a more extensive phylogeographic picture including Andean *Triatoma* species such as *T. maculata* and *T. dispar* should be explored, and additional studies on other factors such as niche differentiation must be considered.

## Supporting Information

Table S1 Additional nucleotide sequences of *T. dimidiata* included in this study.
(DOCX)

Table S2 ITS-2 Haplotype distribution of Colombian *T. dimidiata* used in this study. Map numbers and geographic origin are detailed in Table 1.
(DOCX)

Table S3 ND4 haplotype distribution of *T. dimidiata* used in this study. Map numbers and geographic origin are detailed in Table 1.
(DOCX)
