# Supplementary material for: Molecular Evidence of Demographic Expansion of the Chagas Disease Vector Triatoma dimidiata (Hemiptera, Reduviidae, Triatominae) in Colombia
Source: PLoS Negl Trop Dis. 2014 Mar 13;8(3):e2734. doi: 10.1371/journal.pntd.0002734 (PMC3953067; doi:10.1371/journal.pntd.0002734)
Supplement: Table S1 — Additional nucleotide sequences of T. dimidiata included in this study. (DOCX) [file pntd.0002734.s001.docx]

**Table S1.** Additional nucleotide sequences of *T. dimidiata* included in this study.

| **Marker** | **Number** | **Sequence id.** | **GenBank access** | **Source** |
| --- | --- | --- | --- | --- |
| ITS-2 | 1 | Tdim_H1 | AM286693.1 | Bargues et al., 2008 |
|  | 2 | Tdim_H2 | AM286694.1 |  |
|  | 3 | Tdim_H3 | AM286695.1 |  |
|  | 4 | Tdim_H4 | AM286696.1 |  |
|  | 5 | Tdim_H5 | AM286697.1 |  |
|  | 6 | Tdim_H6 | AM286698.1 |  |
|  | 7 | Tdim_H7 | AM286699.1 |  |
|  | 8 | Tdim_H8 | AM286700.1 |  |
|  | 9 | Tdim_H9 | AM286701.1 |  |
|  | 10 | Tdim_H10 | AM286702.1 |  |
|  | 11 | Tdim_H11 | AM286703.1 |  |
|  | 12 | Tdim_H12 | AM286704.1 |  |
|  | 13 | Tdim_H13 | AM286705.1 |  |
|  | 14 | Tdim_H14 | AM286706.1 |  |
|  | 15 | Tdim_H15 | AM286707.1 |  |
|  | 16 | Tdim_H16 | AM286708.1 |  |
|  | 17 | Tdim_H17 | AM286709.1 |  |
|  | 18 | Tdim_H18 | AM286710.1 |  |
|  | 19 | Tdim_H19 | AM286711.1 |  |
|  | 20 | Tdim_H20 | AM286712.1 |  |
|  | 21 | Tdim_H21 | AM286713.1 |  |
|  | 22 | Tdim_H22 | AM286714.1 |  |
|  | 23 | Tdim_H23 | AM286715.1 |  |
|  | 24 | Tdim_H24 | AM286716.1 |  |
|  | 25 | Tdim_H25 | AM286717.1 |  |
|  | 26 | Tdim_H26 | AM286718.1 |  |
|  | 27 | Tdim_H27 | AM286719.1 |  |
|  | 28 | Tdim_H28 | AM286720.1 |  |
|  | 29 | Tdim_H29 | AM286721.1 |  |
|  | 30 | Tdim_H30 | AM286722.1 |  |
|  | 31 | Tdim_H31 | AM286723.1 |  |
|  | 32 | Tdim_H32 | FJ197146.1 | Dorn et al., 2009 |
|  | 33 | Tdim_H33 | FJ197147.1 |  |
|  | 34 | Tdim_H34 | FJ197148.1 |  |
|  | 35 | Tdim_H35 | FJ197149.1 |  |
|  | 36 | Tdim_H36 | FJ197150.1 |  |
|  | 37 | Tdim_H37 | FJ197151.1 |  |
|  | 38 | Tdim_H38 | FJ197152.1 |  |
|  | 39 | Tdim_H39 | FJ197153.1 |  |
|  | 40 | dzib563 | EF383129.1 | Tamay-Segovia et al. 2008 |
|  | 41 | dzib55 | EF383127.1 |  |
|  | 42 | bolon63 | EF383125.1 |  |
|  | 43 | bolon52 | EF383123.1 |  |
|  | 44 | Dzib62 | EF383128.1 |  |
|  | 45 | bolon504 | EF383126.1 |  |
|  | 46 | becal642 | EF383124.1 |  |
|  | 47 | beca1661 | EF383122.1 |  |
|  | 48 | calak37 | AY860416.1 |  |
|  | 49 | cam4 | AY860414.1 |  |
|  | 50 | cam21 | AY860412.1 |  |
|  | 51 | cam14 | AY860410.1 |  |
|  | 52 | sey43 | AY860408.1 |  |
|  | 53 | calak35 | AY860417.1 |  |
|  | 54 | calak36 | AY860415.1 |  |
|  | 55 | cam6 | AY860413.1 |  |
|  | 56 | cam23 | AY860411.1 |  |
|  | 57 | sey44 | AY860409.1 |  |
|  | 58 | Guatemala_StaAn | DQ871354 | Dorn et al., 007 |
|  | 59 | Guatemala_StaAn | DQ871355 |  |
|  | 60 | Guatemala_StaAn | DQ871356 |  |
|  | 61 | Honduras_SanJo | AJ286875.1 | Marcilla et al., 2001 |
|  | 62 | Mexico_SanLuis | AJ286879.1 |  |
|  | 63 | Mexico_Veracruz | AJ286877.1 |  |
|  | 64 | Mexico_Oaxaca | AJ286878.1 |  |
|  | 65 | Mexico_Yucatan | AJ286880.1 |  |
|  | 66 | Nicaragua_Madriz | AJ286876.1 |  |
| ND4 | 1 | Ec3 | JN620177 | Monteiro et al., 2013 |
|  | 2 | GuVe3 | JN620163 |  |
|  | 3 | Pan3 | JN620175 |  |
|  | 4 | CR5 | JN620174 |  |
|  | 5 | SaLa3 | JN620173 |  |
|  | 6 | HoCa2 | JN620171 |  |
|  | 7 | HoTe3 | JN620172 |  |
|  | 8 | GUA1B | JN620169 |  |
|  | 9 | MxCh3 | JN620159 |  |
|  | 10 | MxCh2 | JN620158 |  |
|  | 11 | TdTHON5 | AF454689 |  |
|  | 12 | TdTHON6 | AF454690 |  |
|  | 13 | TdTHON7 | AF454688 |  |
|  | 14 | TdTHON4 | AF454691 |  |
|  | 15 | TdTHON1 | AF454687 |  |
|  | 16 | TdTGUA1 | AF454692 |  |
|  | 17 | GuVe9 | JN620165 |  |
|  | 18 | GuVe10 | JN620166 |  |
|  | 19 | Gua4 | JN620168 |  |
|  | 20 | GuVe6 | JN620164 |  |
|  | 21 | Gua3 | JN620167 |  |
|  | 22 | CoBy1 | JN620176 |  |
|  | 23 | TdHMEX3 | AF454686 |  |
|  | 24 | TdnVMEX2 | AF454684 |  |
|  | 25 | TdVMEX1 | AF454685 |  |
|  | 26 | MxHi4 | JN620155 |  |
|  | 27 | MxOx3 | JN620156 |  |
|  | 28 | Bz1 | JN620170 |  |
|  | 29 | TdYUC7 | AF454696 |  |
|  | 30 | TdYUC8 | AF454699 |  |
|  | 31 | TdYUC6 | AF454694 |  |
|  | 32 | TdYUC1 | AF454697 |  |
|  | 33 | TdYUC3 | AF454698 |  |
|  | 34 | TdYUC2 | AF454695 |  |
|  | 35 | TdYUC5 | AF454693 |  |
|  | 36 | MxYu10 | JN620160 |  |
|  | 37 | MxCh1 | JN620157 |  |
|  | 38 | GuPe13 | JN620162 |  |
|  | 39 | GuPe12 | JN620161 |  |
