# Supplementary material for: Molecular Evidence of Demographic Expansion of the Chagas Disease Vector Triatoma dimidiata (Hemiptera, Reduviidae, Triatominae) in Colombia
Source: PLoS Negl Trop Dis. 2014 Mar 13;8(3):e2734. doi: 10.1371/journal.pntd.0002734 (PMC3953067; doi:10.1371/journal.pntd.0002734)
Supplement: Table S2 — ITS-2 Haplotype distribution of Colombian T. dimidiata used in this study. Map numbers and geographic origin are detailed in Table 1. (DOCX) [file pntd.0002734.s002.docx]

**Table S2.** ITS-2 Haplotype distribution of *T. dimidiata* of Colombia used in this study. Map numbers and geographic origin are detailed in Table 1.

| **Haplotype code** | **Freq.** | **Origin** | **Boyacá** | | | | | | | | **Huila** | | | **Santander** | **Antioquia** | **Bolívar** | | **Cesar** | | **Córdoba** | **Norte de Santander** | **La Guajira** | | **Magdalena** |
| --- | --- | --- | --- | --- | --- | --- | --- | --- | --- | --- | --- | --- | --- | --- | --- | --- | --- | --- | --- | --- | --- | --- | --- | --- |
| **ITS-2** |  | **Map No.** | **1** | **2** | **3** | **4** | **5** | **6** | **7** | **8** | **9** | **10** | **11** | **12** | **13** | **14** | **15** | **16** | **17** | **18** | **19** | **20** | **21** | **22** |
| Hap_1 | 23 |  |  |  |  | 1 |  |  |  |  |  |  |  | 2 |  | 3 |  |  | 5 | 1 | 4 | 2 | 4 | 1 |
| Hap_2 | 1 |  |  |  |  |  |  |  | 1 |  |  |  |  |  |  |  |  |  |  |  |  |  |  |  |
| Hap_3 | 2 |  |  |  | 1 |  |  |  | 1 |  |  |  |  |  |  |  |  |  |  |  |  |  |  |  |
| Hap_4 | 1 |  |  |  |  |  |  |  | 1 |  |  |  |  |  |  |  |  |  |  |  |  |  |  |  |
| Hap_5 | 1 |  |  |  |  |  |  |  |  | 1 |  |  |  |  |  |  |  |  |  |  |  |  |  |  |
| Hap_6 | 1 |  |  |  |  |  |  |  |  | 1 |  |  |  |  |  |  |  |  |  |  |  |  |  |  |
| Hap_7 | 1 |  |  |  |  |  |  | 1 |  |  |  |  |  |  |  |  |  |  |  |  |  |  |  |  |
| Hap_8 | 1 |  |  |  |  |  |  | 1 |  |  |  |  |  |  |  |  |  |  |  |  |  |  |  |  |
| Hap_9 | 3 |  |  |  |  |  |  |  |  |  |  |  |  |  | 3 |  |  |  |  |  |  |  |  |  |
| Hap_10 | 1 |  |  |  |  |  |  |  |  |  |  |  |  |  |  |  |  | 1 |  |  |  |  |  |  |
| Hap_11 | 1 |  |  |  |  |  |  |  |  |  |  |  |  |  |  |  |  | 1 |  |  |  |  |  |  |
| Hap_12 | 1 |  |  |  |  |  |  |  |  |  |  |  |  |  |  |  |  |  |  | 1 |  |  |  |  |
| Hap_13 | 1 |  |  |  |  |  |  |  |  |  |  |  |  |  |  |  |  |  |  |  |  |  |  | 1 |
| Hap_14 | 1 |  |  |  |  |  |  |  |  |  |  |  |  |  |  |  |  |  |  |  | 1 |  |  |  |
| Hap_15 | 1 |  |  |  |  |  |  |  |  |  |  |  |  |  |  |  |  |  |  |  | 1 |  |  |  |
| Hap_16 | 1 |  |  |  |  |  |  |  |  |  |  |  |  |  |  |  |  |  |  |  | 1 |  |  |  |
| Hap_17 | 1 |  |  |  |  |  |  |  |  |  |  |  |  | 1 |  |  |  |  |  |  |  |  |  |  |
